# Phylogenetic approaches resolve taxonomical confusion in *Pedicularis* (Orobanchaceae): Reinstatement of *Pedicularis delavayi* and discovering a new species *Pedicularis milliana*

**DOI:** 10.1371/journal.pone.0200372

**Published:** 2018-07-25

**Authors:** Wen-Bin Yu, Hong Wang, Min-Lu Liu, Alisa E. Grabovskaya-Borodina, De-Zhu Li

**Affiliations:** 1 Center for Integrative Conservation, Xishuangbanna Tropical Botanical Garden, Chinese Academy of Sciences, Mengla, China; 2 Southeast Asia Biodiversity Research Institute, Chinese Academy of Science, Yezin, Nay Pyi Taw, Myanmar; 3 Key Laboratory for Plant Diversity and Biogeography of East Asia, Kunming Institute of Botany, Chinese Academy of Sciences, Kunming, China; 4 Plant Germplasm and Genomics Center, Germplasm Bank of Wild Species, Kunming Institute of Botany, Chinese Academy of Sciences, Kunming, China; 5 Komarov Botanical Institute, Prof. Popov str., St. Petersburg, Russia; College of Agricultural Sciences, UNITED STATES

## Abstract

Morphological identification of *Pedicularis* depends on floral characters. However, some important characters may be lost during the process of pressing the specimen. *Pedicularis delavayi* was described from northwestern Yunnan, and widely adopted as a variety of *P*. *siphonantha*. Unfortunately, the name “*P*. *siphonantha* var. *delavayi*’ incorrectly referred to *P*. *milliana* (a new species described in this study) or *P*. *tenuituba* in some herbarium specimens and publications. Moreover, phylogenetic relationships among *P*. *delavayi*, *P*. *siphonantha* and its allies (*P*. *milliana* and *P*. *tenuituba*) were not fully resolved. In this study, we sampled 76 individuals representing 56 taxa. Of them, 10 taxa were from *P*. *siphonantha* lineage, and 11 individuals of *P*. *delavayi* represented 9 populations. These species were named as *P*. *siphonantha* group on the basis of morphological similarity. Nuclear ribosomal internal transcribed spacer (nrITS) and four chloroplast genes/regions were used for phylogenetic analyses. Phylogenetic analyses showed that the *P*. *siphonantha* group was polyphyletic: *P*. *delavayi* was sister to *P*. *obliquigaleata* in clade A; and the remaining species of *P*. *siphonantha* group were monophyletic in clade B, named as *P*. *siphonantha* lineage. In the *P*. *siphonantha* lineage, *P*. *milliana*, *P*. *siphonantha*, and *P*. *tenuituba* were well supported as monophyletic, and *P*. *dolichosiphon* was sister to *P*. *leptosiphon*. Morphologically, *P*. *delavayi* differs from species of the *P*. *siphonantha* lineage in having a long petiole (~ 50 mm) and pedicel (~ 40 mm), a ridged corolla tube, and a folded lower-lip of the corolla. Therefore, both morphological characters and phylogenetic evidence strongly supported to reinstate *P*. *delavayi* as an independent species and describe *P*. *milliana* as new species. In addition, *P*. *neolatituba* was proposed to reduce as a new synonymy of *P*. *delavayi*.

## Introduction

Flowers of *Pedicularis* L. (Orobanchaceae) show striking interspecific variations [1, 2], so morphological identification of these species depends much on floral characters [3]. Generally, one species is easily distinguished from another morphologically similar species using fresh flowers in the field. However, floral shape and structure may be changed during the process of pressing and drying the specimen and, in practice, herbarium specimens of closely related species are very difficult to discriminate. DNA barcodes have been widely applied to assist species identification [4–6], particularly when morphological identification is uncertain. In *Pedicularis*, the nuclear ribosomal internal transcribed spacer (nrITS) or nrITS+*rbcL* can discriminate at least 78% of species in the genus [3, 7]. However, there is little consensus between the phylogenetic tree and traditional classification in *Pediculari*s [8–12], and morphologically similar species may not be sister to each other in phylogenetic analyses. Therefore, DNA sequences are very useful to delimit species and to confirm phylogenetic relationship among species.

**Fig 1:**
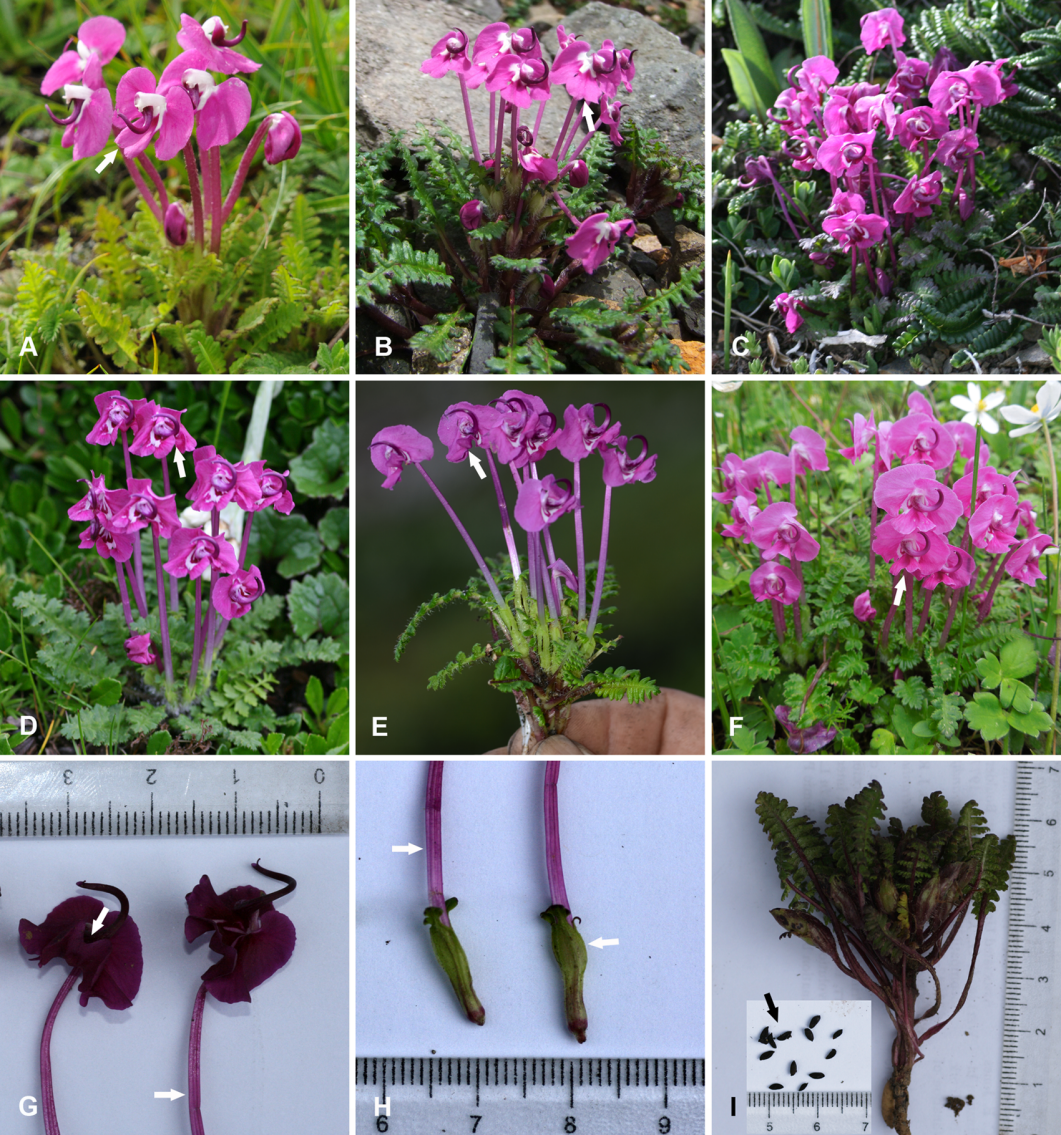
Field photos of *P*. *delavayi* Franch. ex Maxim., *P*. *milliana* W. B. Yu, D. Z. Li & H. Wang and *P*. *siphonantha* D. Don. A, *P*. *siphonantha*. B, *P*. *milliana*. C-I, *P*. *delavayi*: C, G-I, from Daxue Mtn.; D from Hong Mtn.; E from Yulong Mtn.; F from Wuxu Lake. A spreading middle lobe of the corolla lower-lip with emargination indicated by an arrow in A and B; a folded middle lobe of the corolla lower-lip with emargination indicated by an arrow in D-G; a ridged corolla tube indicated by an arrow in G and H; an inflated calyx tube in the middle upper parts indicated by an arrow in H; black seeds indicated by an arrow in I. A and B were taken by Z.-K. Wu; E by H.-D. Li; C, D, and F-I by W.-B. Yu.

*Pedicularis delavayi* was firstly named by Franchet after J. M. Delavay, who collected the type material (S1 Fig) from Yulong Mountain in Lijiang, northwestern Yunnan, China in 1886, while it was validly published by Maximowicz [13]. This species was treated as an independent species [14–17] until Tsoong [18], who downgraded it to a variety in *P*. *siphonantha* D. Don. According to illustrations in Chinese Floras [18, 19], the lower lip of *P*. *siphonantha* var. *delavayi* (Franch. ex Maxim.) P. C. Tsoong should be similar to *P*. *siphonantha* var. *siphonantha* as spreading (see Fig 1A). From illustrated publications, the name *“P*. *siphonantha* var. *delavayi* was used for a “long-tubed and purple-red species” (Fig 1B), which is a common species in alpine meadow at altitudes from 3000 m to 4000 m a.s.l. (above sea level, hereafter) in northwestern Yunnan [20–22]. In addition, some publications just used the name “*P*. *siphonantha*” referring to this “long-tubed and purple-red species” in northwestern Yunnan [23–27].

During field expeditions for *Pedicularis* in the Hengduan Mountains region from 2006 to 2010, we collected an unknown long-tubed species with a purple-red corolla in Shangri-La, northwest Yunnan, and in Jiulong and Kangding, Sichuan, at altitudes around 4000 m (Fig 1C–1F). This species differs from infraspecfic taxa of *P*. *siphonantha* and other long-tubed and purple-red species in series *Longiflorae* Prain by having a folded middle lobe of the lower lip (Fig 1C–1G) and a ridged corolla tube (Fig 1G and 1H). DNA barcoding showed that samples of this species were separated from *P*. *siphonantha* [7]. We considered that this species may be new until we checked the type materials of *P*. *delavayi* conserved at the herbaria of the V. L. Komarov Botanical Institute in St. Petersburg (LE) (S1 Fig) and the Muséum National d’Histoire Naturelle in Paris (P). Based on morphological comparisons of specimens, we found that our specimens were very similar to the type of *P*. *delavayi*. In order to clarify the taxonomical confusion, we examined herbarium specimens of *P*. *siphonantha* collected from Yulong Mountain (type location of *P*. *delvayi*) conserved at the herbaria of CAS Kunming Institute of Botany (KUN) and CAS Institute of Botany (PE). We found that specimens labeled as “*P*. *siphonantha* var. *delavayi*” included two taxa: one is similar to the type of *P*. *delavayi*, and another is the “long-tubed and purple-red species” (Fig 1B), a common species in northwestern Yunnan. Indeed, it is very difficult to discriminate the herbarium specimens as two taxa. Based on the field investigations, we found that *P*. *delavayi* differed from the “long-tubed and purple-red species” by having a long petiole and pedicel, inflated calyx tube in the middle upper parts, and folded lower lip of the corolla, as well as occurring at altitudes over 3600 m a.s.l. During recent field expeditions, specimens of *P*. *delavayi* were collected from the Yulong Mountain at over 4000 m a.s.l (Fig 1F), and those of the “long-tubed and purple-red species” between 3600 m and 4000 m a.s.l. (Fig 1B). Therefore, we confirmed that *P*. *delavayi* and the “long-tubed and purple-red species” were two separated species, and the “long-tubed and purple-red species” should be an undescribed species. In this study, we proposed and described the “long-tubed and purple-red species” as a new species *P*. *milliana* W. B. Yu, D. Z. Li & H. Wang.

A comprehensive phylogeny of Chinese *Pedicularis* shows that the species *P*. *siphonantha* is a polyphyletic group, var. *delavayi* (≡ *P*. *delavayi*) and other varieties of *P*. *siphonantha* falling into two subclades in clade 3 [8]. *Pedicularis delavayi* was sister to *P*. *obliquigeleata* in subclade A. In the subclade 3B, var. *siphonantha* and var. *stictochila* H. Wang & W.B. Yu (= *P*. *tenuituba* H.L. Li), and five species from series *Longiflorae* by having purple/red/pink corollas with twisted beaks formed a strongly supported lineage, i.e. the *P*. *siphonantha* lineage, which is a molecular delimitation. In the present study, we made extensive sampling of *P*. *delavayi* from nine populations (10 new samples), and two additional samples of *P*. *milliana* from Yulong Mountains in Lijiang, one additional new sample of *P*. *tenuituba*, and selected taxa from all recognized monophyletic lineages in clade 3, and the sister *P*. *axillaris* Franch. ex Maxim. [8]. DNA sequences from nrITS and four chloroplast regions (*matK*, *rbcL*, *trnH-psbA* and *trnL-F*) were generated and analyzed. Our main goal was to evaluate the monophyly of *P*. *delavayi*, and its phylogenetic relationship to the *P*. *siphonantha* lineage, in particular to *P*. *milliana* and *P*. *tenuituba*. If the monophyletic *P*. *delavayi* was excluded from the *P*. *siphonantha* lineage, *P*. *delavayi* should be reinstated as an independent species; and the monophyly of *P*. *milliana* was supported, which should be described and illustrated as a new species.

## Material and methods

### Ethics statement

No specific permissions were required for these locations/activities during the fieldwork, and none of studied species was listed as endangered or protected species in the first batch of “China’s Catalogue of the National Protected Key Wild Plants” (http://www.forestry.gov.cn/yemian/minglu1.htm).

### Plant samplings

In total, we sampled 76 individuals representing 56 taxa, including all representative taxa were identified in clade 3, and its sister *P*. *axillaris* [8]. *Pedicularis siphonantha* group, based on morphological delimitation, consisted of 11 species (S1 Table). Of them, four species *P*. *delavayi*, “*P*. *milliana*” (an undescribed species), *P*. *siphonantha*, and *P*. *tenuitub*a (= *P*. *siphonantha* var. *stictochila*) have a wide distribution range. The remaining seven species only collected once or few gatherings around the type locality: 1) *P*. *sigmoidea* Franch. ex Maxim. were found in Eryuan and Lijiang, northwest Yunnan; 2) *P*. *dolichosiphon* (Hand.-Mazz.) H.L.Li) (≡ *P*. *siphonantha* var. *dolichosiphon* Hand.-Mazz.), *P*. *dolichantha* Bonati, *P*. *leptosiphon* H. L. Li, *P*. *variegata* H. L. Li and *P*. *humilis* Bonati were recollected from the type locality; and 3) *P*. *fastigiata* Franch. only had the type, which was not included in this study. In this study, we chose 11 samples of *P*. *delavayi* (three samples from the type locality Yulong Mountain, Lijiang), and three samples of *P*. *milliana* and *P*. *tenuituba* (S2 Table). Natural population of *P*. *humilis* was just rediscovered in 2015 [28]. It is the first time to include this species for phylogenetic analyses.

Fresh leaf tissues were collected in the field and preserved in silica gel. All DNA samples and voucher specimens are stored at the Germplasm Bank of Wild Species and the herbarium of CAS Kunming Institute of Botany (KUN), respectively. There are 284 sequences from 64 individuals which have been published in other studies [6–8, 29]. In this study, we generated 62 new sequences from 23 individuals (with 11 newly sampled individuals). A conspectus of voucher information is presented in S2 Table. The DNA sequence matrix is available in S1 File.

### Specimen examination and identification

Fresh specimens were observed in the field. Fresh flowers were collected and fixed in FAA solution. Herbarium specimens from the herbaria CDBI, KUN, LE, MPU, and PE were examined and identified, and digital images of types from the herbaria E, K and P were accessed online. Flower and fruits characters in the line drawings of *P*. *delavayi* were based on field photos and FAA-preserved flowers.

### DNA isolation, PCR and sequencing

For the 11 new samples, total genomic DNA was extracted from silica gel-dried tissue using a modified 2× CTAB method. Five DNA loci, one nuclear region (nrITS) and four chloroplast genes/regions (*matK*, *rbcL*, *trnH-psbA*, and *trnL-F*), were sequenced in this study. Primer information for the five loci were presented in previous studies [7, 30]. Protocols for polymerase chain reaction (PCR) amplification and sequencing followed the study of Yu et al. [7].

### Sequence assembly and alignment

The newly obtained raw sequences were assembled and edited using Geneious version 7.1 [31]. The nrITS is a multiple copy region. These copies showed evolutionary consistent in the sequenced 75 samples, only one sample, HW10244 belonging to *P*. *tenuituba*, had one ambiguous basecall (i.e. multiple superimposed peaks in chromatograms). The ambiguous site was assigned using IUPAC ambiguity characters.

Preliminary alignments were automatically aligned using MAFFT version 7.2 [32], then adjusted manually in Geneious. The aligned matrix was concatenated to a combined matrix using SequenceMatrix version 1.73 [33]. Sequence characteristics were calculated using MEGA version 6.0 [34].

### Phylogenetic analyses

Bayesian Inference (BI) and Maximum Likelihood (ML) methods were used to reconstruct phylogenetic trees. The nrITS and plastid datasets were combined to analyze. No nucleotide positions were excluded from analyses. Partitioned BI analyses were performed using MrBayes [35], with DNA substitution models selected for each gene partition by the Bayesian information criterion (BIC) using jModeltest [36, 37]. Markov Chain Monte Carlo (MCMC) analyses were performed using MrBayes for 10,000,000 generations for the dataset, with two simultaneous runs, and each run comprising four incrementally heated chains. The BI analyses were started with a random tree and sampled every 1000 generations. Number of generations for the dataset were sufficient, because the average standard deviation of split frequencies for the dataset was lower than 0.005 (0.002900), and Potential Scale Reduction Factor of Convergence Diagnostic [38] for the datasets was 1.00. The first 25% of the trees was discarded as burn-in, and the remaining trees were used to generate a majority-rule consensus tree. Posterior probability values (PP) ≥ 0.95 were considered as well supported [39–41]. The ML tree searches and bootstrap estimation of clade support were conducted with RAxML [42]. These analyses used the GTR substitution model with gamma-distributed rate heterogeneity among sites and the proportion of invariable sites estimated from the data. The dataset was partitioned by genes. Support values for the node and clade were estimated from 1000 bootstrap replicates. Bootstrap support (BS) ≥ 70 are considered well supported [43]. Both BI and ML analyses, as well as jModelTest, were performed at the CIPRES Science Gateway (http://www.phylo.org).

### Nomenclature

The electronic version of this article in Portable Document Format (PDF) in a work with an ISSN or ISBN will represent a published work according to the International Code of Nomenclature for algae, fungi, and plants [44], and hence the new names contained in the electronic publication of a PLOS ONE article are effectively published under that Code from the electronic edition alone, so there is no longer any need to provide printed copies.

In addition, new names contained in this work have been submitted to IPNI, from where they will be made available to the Global Names Index. The IPNI LSIDs can be resolved and the associated information viewed through any standard web browser by appending the LSID contained in this publication to the prefix http://ipni.org/. The online version of this work is archived and available from the following digital repositories: PubMed Central (https://www.pubmedcentral.nih.gov/) and Researchgate (https://www.researchgate.net/)

## Results

### Information of DNA sequences

Sequence characteristics of five DNA regions and the concatenated datasets are summarized in Table 1. In the datasets, the numbers of variable and parsimony informative sites were highest for nrITS, followed by *trnH-psbA*, *trnL-F*, *matK* and *rbcL*. For three selected groups (*P*. *delavayi*, *P*. *siphonantha* lineage, and *P*. *delavayi* + *P*. *siphonantha* lineage, i.e. *P*. *siphonantha* group), three spacers (nrITS, *trnH-psbA* and *trnL-F*) were more variable and informative than two coding genes (*matK* and *rbcL*), then *matK* was more than *rbcL*. One exception for *P*. *delavayi*, the alignment of *matK* had only one variable site in the 11 individuals, whereas alignment of *rbcL* had three variable and two informative sites, respectively.

**Table 1:** Sequence characteristics of nrITS and four plastid DNA regions.

Parameters	nrITS	Plastid genes				Total dataset
		*matK*	*rbcL*	*trnH-psbA*	*trnL-F*	
**No. of accessions**	75	73	72	52	74	75
**Aligned length (bp)**	625	705	624	644	1045	3643
**Variable sites/Parsimony informative sites**						
**Total dataset**	211/144	161/85	60/37	200/89	234/108	866/463
***P*. *delavayi* + *P*. *siphonantha* lineage**	65/33	52/29	19/14	78/37	63/33	277/146
***P*. *delavayi***	5/1	1/0	3/2	6/2	6/4	21/9
***P*. *siphonantha* lineage**	52/21	42/20	15/11	67/30	52/22	228/104

### Phylogenetic analyses

**Fig 2:**
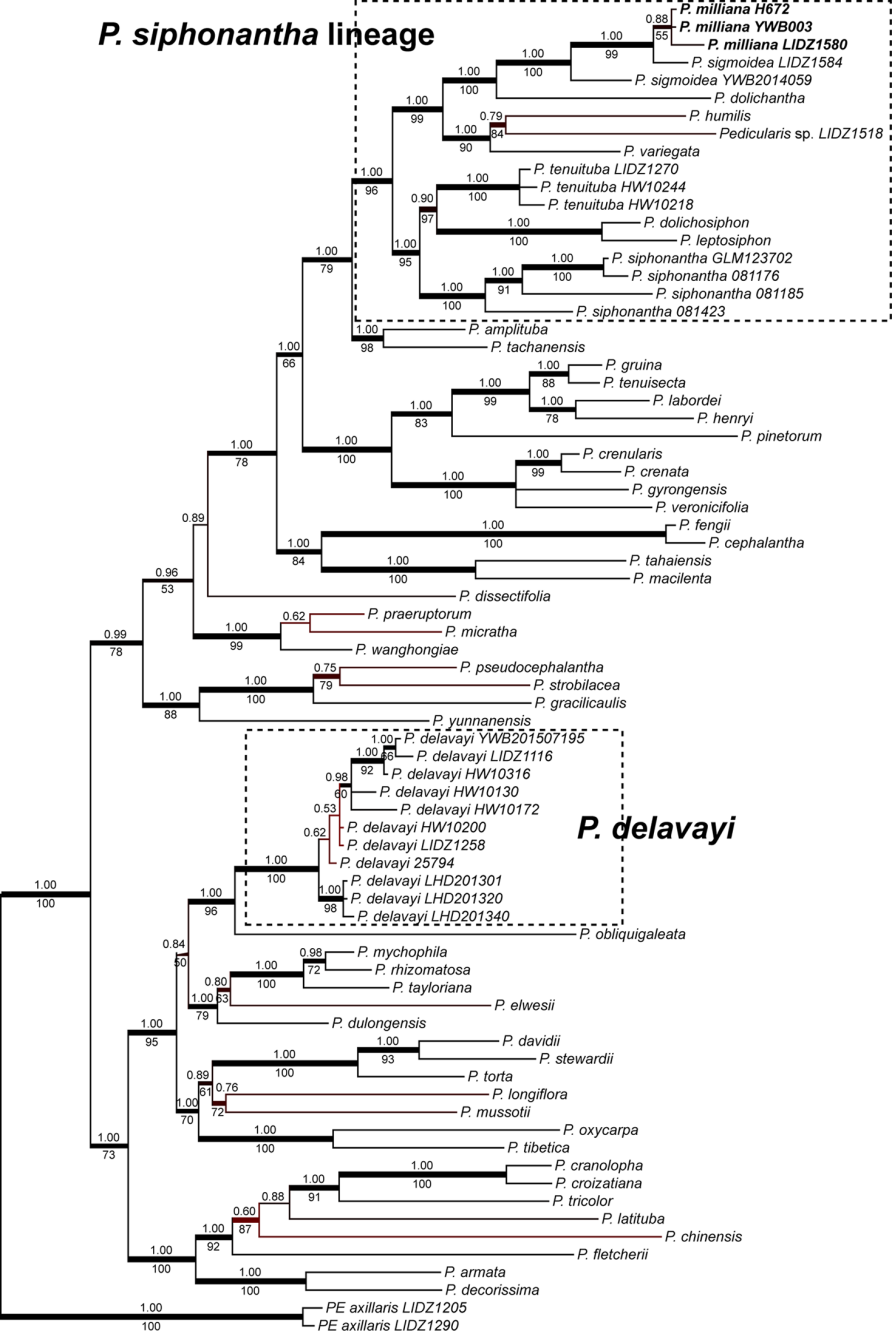
Phylogeny of the *Pedicularis siphonantha* group inferred from Bayesian Inference (BI) and Maximum Likelihood (ML) methods using the combination of nuclear ribosomal internal transcribed spacer (nrITS) and four plastid (*matK*, *rbcL trnH-psbA* and *trnL-F*) datasets. Topology shows the majority rule consensus of the BI tree. BI posterior probability (PP) ≥ 0.50 and ML bootstrap support (BS) ≥ 50 were annotated on the branch. PP ≥ 0.95 and/or BS ≥ 70 were drawn with thicker and black lines.

The BI tree using the total dataset is presented in Fig 2. The topology was similar to that in previous study [8]. Two major clades were recovered, named as A and B following Yu et al. [8]. *Pedicularis delavayi* fell into clade A, and *P*. *siphonantha* lineage was in clade B. Both *P*. *delavayi* (BS/PP = 100/1.00) and *P*. *siphonantha* lineage (BS/PP = 96/1.00) were strongly supported as monophyletic, respectively. In the clade of *P*. *delavayi*, three Lijiang samples (from the type locality) formed a group (BS/PP = 98/1.00), which was weakly supported sister to the remaining eight samples (PP = 0.62); three Sichuan samples were strongly supported as monophyletic (BS/PP = 92/1.00), and two Yunnan samples (HW10130 and HW10172) as sister. The *P*. *siphonantha* lineage split in two groups. One group included clade *P*. *dolichosiphon* + *P*. *leptosiphon* (BS/PP = 100/1.00), and monophyletic *P*. *siphonantha* (BS/PP = 100/1.00) and *P*. *tenuituba* (= *P*. *siphonantha* var. *stictochila*) (BS/PP = 100/1.00). Another group comprised of the remaining five sampled species (including *P*. *humilis*) and sample LIDZ1518. Three samples of *P*. *milliana* from Lijiang were monophyletic by moderate supporting (BS/PP = 55/0.88), then the Lijiang sample (LIDZ1584) of *P*. *sigmoidea* was resolved as sister (BS/PP = 99/1.00), followed by the Eryuan sample (YWB2015059) of *P*. *sigmoidea* (BS/PP = 100/1.00). *Peducularis humilis* nested with sample LIDZ1518 (BS/PP = 84/0.79), with long branch length, and *P*. *variegata* was sister to them (BS/PP = 90/1.00).

### Morphological comparisons

Morphologically, *P*. *delavayi* is similar to *P*. *siphonantha* by having large and bi-lobed middle lobe of lower-lip, and semi-circle and crestless beak. However, the middle lobe of *P*. *delavayi* was significantly incurved (Fig 1C–1G; vs. spreading in *P*. *siphonantha*, Fig 1A), which was crushed in herbarium specimens (e.g. S1 Fig). Based on comparisons of flowering specimens, we found that *P*. *delavayi* also differed from *P*. *siphonantha* by having a long petiole (~ 50 mm) and pedicel (~ 40mm), a furfuraceous surface on the abaxial leaf blade, a ridged corolla tube, a folded lower-lip of the corolla, and four pubescent filaments (S2 Fig). In addition, we found that the type of *P*. *neolatituba* (type specimen online: http://pe.ibcas.ac.cn/en/) was very close to specimens of *P*. *delavayi* and should be reduced to a synonym of *P*. *delavayi*.

*Pedicularis milliana* (Fig 1C) is very similar to *P*. *siphonantha* by having smooth corolla tube, spreading corolla lower-lip, large and bi-lobed middle lobe, and semi-circle and crestless beak. Because the distribution of *P*. *delavayi* overlaps with *P*. *milliana* in northwestern Yunnan, Tsoong [18] might consider the plants of *P*. *milliana* as *P*. *delavayi*. Therefore, he downgraded *P*. *delavayi* to a variety under *P*. *siphonantha*. Clearly, *P*. *milliana* was separated from *P*. *siphonantha*, which is strongly supported by phylogenetic analyses. In addition, sample LIDZ1518 (an unknown taxon) was similar to *P*. *milliana* in having spreading corolla lower-lip, large and bi-lobed middle lobe, and semi-circle beak, and to *P*. *sigmoidea* in having spreading corolla lower-lip, large and bi-lobed middle lobe, and crested beak. However, phylogenetic analyses indicating it was a separated lineage, close to *P*. *humilis* and *P*. *variegata*.

## Discussion

### Phylogenetic delimitation of *P*. *siphonantha* group

*Pedicularis siphonantha* was firstly described from Nepal [45], which has been recognized as endemic to the Himalayan region [17, 46]. According to current taxonomic treatments [18, 19], *P*. *siphonantha* var. *delavay* and var. *stictochila* occur in the Hengduan Mountains region, i.e. northwestern Yunnan, western Sichuan, and southeastern Qinghai. *Pedicularis siphonantha* var. *dolichosiphon* was discovered in Muli, south Sichuan [47], then upgraded to an independent species by Li [17]. In the Chinese edition of *Flora Reipublicae Popularis Sinicae*, Tsoong [18] did not mention *P*. *dolichosiphon*, or he might have overlooked this species. According to current phylogenetic analyses, *P*. *siphonantha* was polyphyletc, delimitation of *P*. *siphonantha* group needed to revise. Firstly, *P*. *siphonantha* var. *delavayi* was close to *P*. *obliquigaleata* in clade A, whereas the other taxa of *P*. *siphonantha* were included in the *P*. *siphonantha* lineage. Therefore, *P*. *siphonantha* var. *delavayi* should be reinstated as an independent species. Then, the remaining three infraspecific taxa of *P*. *siphonantha* (var. *dolichosiphon*, var. *siphonantha* and var. *stictochila*) and *P*. *leptosiphon* formed a clade, and var. *dolichosiphon* was strongly supported as sister to *P*. *leptosiphon*. Of them, *P*. *siphonantha* var. *siphonantha* has a semicircle beak, and the other three taxa have S-shaped beak. Integrating geographical distribution, we agree with the treatment by Li [17] to adopt var. *dolichosiphon* and var. *stictochila* as independent species as *P*. *dolichosiphon* and *P*. *tenuituba*, respectively.

Infraspecific delimitation of *P*. *siphonantha* was not fully resolved. In a taxonomical revision, Prain [16] included *P*. *hookeriana* Wall. ex Benth. as a synonym of *P*. *siphonantha* var. *siphonantha*, and *P*. *elephas* Boiss. and *P*. *punctata* Decne. as synonyms of var. *brevituba* Prain. Nevertheless, some taxonomists treated *P*. *hookeriana* and *P*. *punctata* as independent species [48–50], and have placed *P*. *elephas* close to *P*. *rhinanathoides* Schrenk [13, 48]. A comprehensive phylogeny of *Pedicularis* showed that both *P*. *hookeriana* and *P*. *punctata* fell into the clade of *P*. *siphonantha* from the Himalayas (R. Ree, Personal Communication). In the early of 1900s, Bonati added two varieties under *P*. *siphonantha*, var. *prostrata* Bonati [51] from Sikkim and var. *birmanica* Bonati [52] from upper Burma. In a revision of *Pedicularis* from Bhutan, Mill [53] pointed out that *P*. *siphonantha* var. *prostrata* was easily confused with *P*. *hookeriana*, whereas this variety had broader and ovate leaves and shorter corolla tubes. For *P*. *siphonantha* var. *birmanica*, we found its type materials were close to that of *P*. *humilis*. Therefore, *P*. *siphonantha* var. *birmanica* should be reduced as a synonym of *P*. *humilis*.

### Parallel evolution of long-tubular corollas in *Pedicularis*

During revision of *Pedicularis*, Li [2, 17, 54] and Tsoong [18, 55] hypothesized that long-tubular corollas were independently evolved at least six and ten times, respectively. Phylogenetic inferences supported their hypotheses that long-tubular corollas were independently derived from short-tubular corollas at least eight times [9], or up to 21 times [8]. Long-tubular species occurred in seven of 13 clades, plus two unresolved species *P*. *batangensis* Franch. & Bur. and *P*. *flexuosa* Hook. f. [8]. Series *Longiflorae* Prain included more than 20 long-tubular species from the Himalaya-Hengduan Mountains region [17, 18, 49, 53]. Species of series *Longiflorae* fell into clade 3, however, this series was not supported as monophyletic (see Results in [8], and this study). Phylogenetic analyses tended to split series *Longiflorae* into four groups: a) *P*. *siphonantha* lineage, b) *P*. *delavayi*, c) *P*. *longiflora*, and 4) *P*. *armata*–*P*. *cranolopha* group (including a short-tubular species, *P*. *fletcherii*). From morphological similarity and geographical distribution, *P*. *delavayi* and *P*. *longiflora* were close to *P*. *siphonantha* lineage and *P*. *armata*–*P*. *cranolopha* group, respectively. However, phylogenetic evidence indicated that the four groups may evolve independently.

Evolution of long-tubular corollas in *Pedicularis* were hypothesized to adopt long-tongued pollinators [2]. However, pollination observations showed that long-tubular species were exclusively pollinated by bumblebees [23, 24, 56–59]. Long-tubular corollas are associated with beaked galea, and beaked species rewards pollinators for pollen only [1, 56]. Due to anthers are tightly enclosed by the beaked galea, long-tongued Lepidoptera are impossible to dislodge pollen from the tightly enclosed anthers. Only bumblebees can open the concealed anthers from the beaked galea using forelegs, and release pollen by vibrating wings in high speed, i.e. buzz-pollination [60]. When long-tongued pollinators driving evolution of long-tubular corollas was rejected, an alternative hypothesis for enhancing pollination attractiveness was proposed [56, 58]. However, pollinator attraction hypothesis was not supported by experiments on *P*. *siphonantha* (corrected as *P*. *milliana* herein) and *P*. *tricolor* Hand.-Mazz. [24]. Pollination treatments indicated that elongation of corolla tube (and pistil length) may put more selective pressure for male-to-male competition during the pollen germination [61]. Moreover, plants growing in more fertilized conditions can produce longer corolla tube [24]. We suggested that evolution of long-tubular corollas may have some advantages in high altitudes, because most of long-tubular species occur in alpine meadow over 3000 m a.s.l. in the Himalya-Hengduan Mountains region [62, 63]. Such ecological factors may independently drive elongation of corolla tube in different lineages. Subsequent diversification of lineage may be mainly induced by geographical isolation. *Pedicularis siphonantha* lineage is one good example to illustrate geographical isolation facilitating species divergence in the Himalya-Hengduan Mountains region [8].

### Reinstatement of *Pedicularis delavayi*

Phylogenetic analyses strongly support *P*. *delavayi* as a separated species, which is sister to *P*. *obliquigaleata* in clade A, not included in *P*. *siphonantha* lineage in clade B. From floral color and beak shape, *P*. *delavayi* was easy to misplace into the *P*. *siphonantha* group. In the revision of Chinese *Pedicularis*, Li [17] cited dozens of specimens for *P*. *delavayi*; however, some Sichuan specimens were *P*. *tenuituba*, and some Yunnan specimens were *P*. *milliana*. Subsequently, Tsoong [18] might be failed to check diagnostic characters of *P*. *delvayi*, or might misplaced the plants of *P*. *milliana* or *P*. *tenuituba* as *P*. *delavayi*, thus he downgraded *P*. *delavayi* as a variety in *P*. *siphonantha*. Unfortunately, Tsoong’s incorrect treatment has been widely adopted by current Chinese Floras [19, 64], checklists [20, 21, 65] and other publications [22, 66]. Moreover, illustrations and/or voucher specimens of “*P*. *siphonantha* var. *delvayi*” from northwestern Yunnan were *P*. *milliana*, or mixed with *P*. *milliana* [20, 21, 22, 65, 66]. Some herbarium specimens of *P*. *tenuituba* from Sichuan were misidentified as “*P*. *siphonantha* var. *delvayi*”. According to morphological and phylogenetic evidence, we propose to reinstate *P*. *delavayi* as an independent species. Full description and line drawing (see S2 Fig) were provided.

*Pedicularis neolatituba* P. C. Tsoong was described from Songpan, northern Sichuan, which had short plant (less than 10 cm), long pedicel (up to 40 mm) and basal circinate-incurved galea [18]. In protologue, Tsoong proposed this species similar to three long-pedicelled species, *P*. *franchetiana*, *P*. *mussotii*, and *P*. *mychophila*, then established series *Neolatitubae* P. C. Tsoong. After checking the type specimen of *P*. *neolatituba*, we found that it was difficult to distinguished from specimens of *P*. *delavayi*. *Pedicularis delavayi* also has long pedicel, anterior cleft and mid-upper part inflated calyx, basal twisted galea, semi-circle beak, ciliate corolla lobes and pubescent filaments. The plant height is variable in different specimens. Therefore, we proposed to reduce *P*. *neolatituba* as a new synonymy of *P*. *delavayi*.

### Delimitation of new species *P*. *milliana*

The new species, *P*. *milliana*, is a common meadow species at altitudes between 3000 m and 4000 m in northwestern Yunnan, where it overlaps with *P*. *delavayi*. Because previous revisions of Chinese *Pedicularis* by Li [17] and Tsoong [18] were mainly based on herbarium specimens, the flat and dry flowers made these authors overlook the fact that *P*. *delavayi* bore a folded lower corolla lip, so specimens of *P*. *milliana* were treated as *P*. *delavayi*. Therefore, the altitudinal range of *P*. *delavayi* was described as from 3000 m to 4600 m a.s.l. [17–19]. Based on field investigations, we found that *P*. *delavayi* only grew at altitudes above 4000 m a.s.l. in northwestern Yunnan, and could extend to 3600 m a.s.l. in Jiulong, western Sichuan. By contrast, *P*. *milliana* preferred growing at altitudes between 3000 m and 3800 m a.s.l. in northwestern Yunnan. In addition, the habitat of *P*. *delavayi* is dry meadow or low shrub, while that of *P*. *milliana* is moist meadows or wetland margins. Taxonomic confusion between *P*. *delavayi* and *P*. *milliana* has mainly been caused by the loss of key morphological characters in dried specimens, whereas fresh floral characters easily distinguish *P*. *milliana* from *P*. *delavayi*. Phylogenetic analyses supported *P*. *milliana* sister to *P*. *sigmoidea*. To further clarify phylogenetic relationship between *P*. *milliana* and *P*. *sigmoidea* needs to extensively sample more populations of them in northwestern Yunnan. It is noteworthy that phylogenetic analysis is an effective approach to delimit the new species *P*. *milliana*, and to resolve its phylogenetic placement.

In the *P*. *siphonantha* lineage, the floral shape of *P*. *milliana* is similar to *P*. *siphonantha* in tube length, semicircle twisted beak, and sub-equal lobes and emarginate mid-lobe of lower lip. However, phylogenetic analyses showed that *P*. *milliana* fell into the clade with *P*. *dolichantha*, *P*. *humilis*, *P*. *sigmoidea*, *P*. *variegata*, and unknown taxon. Specimen collections show that *P*. *dolichantha* was only collected from the type locality Huize, northeast Yunnan; *P*. *humilis* is rediscovered in the type locality at the south Gaoligong Mountain, west Yunnan; *P*. *sigmoidea* is restricted to Dali, Lijiang and Heqing, northwest Yunnan; *P*. *variegata* is only found in Muli, southwest Sichuan, and the unknown taxon is collected in Jiaozi Mountain, north-central Yunnan. Phylogenetic relationships and geographic patterns indicate that evolution of *P*. *milliana* and its relatives from the southwestern mountains of China should be independent from the Himalayan *P*. *siphonantha*. Therefore, the geographical barrier created by high mountains in southwestern China may have facilitated species divergence among *P*. *milliana* and its relatives. *Pedicularis milliana* is a new species which is uncovered by both morphological characters and DNA sequences.

### Taxonomic treatment

*Pedicularis delavayi* Franch. ex Maxim. [urn:lsid:ipni.org:names:806977–1], Bull. Acad. Imp. Sci. Saint-Pétersbourg 32: 531, pl. 1, fig. 7. 1888 ≡ *Pedicularis siphonantha* var. *delavayi* (Franch. ex Maxim.) P. C. Tsoong, Fl. Reipubl. Popularis Sin. 68: 374. 1963. Type: China. Yunnan: Lijiang (Li-kiang), Yulong Snow Mountain (Suee Shan), alt. 4,000m, 14 Aug. 1886, *J*. *M*. *Delavay s*.*n*. (holotype, LE!, barcode 01010308; isotypes, K!, barcode 000708729, MPU!, barcode 020765, P!, barcode 02987194).

*Synonymy*: *Pedicularis neolatituba* P. C. Tsoong [urn:lsid:ipni.org:names:807391–1, misspelled as “neolatimba” in IPNI], in Fl. Reipubl. Popularis Sin. 68: 418–419, pl. 72, f. 1–3. 1963. Syn. nov. Type: China. Sichuan: Songpan (Dongrergo), alt. 4,700m, 9 Aug 1922, *H*. *Smith 3162* (holotype, PE!, barcode 00033070; isotype: PE!, barcode 00119661).

Perennial herb, barely 10 cm tall, drying black or not. Roots fleshy, fusiform. Stems 1 to several, unbranched and erect or ± ascending, 2–10 cm, with lines of hairs. Basal leaves numerous, mostly membraneous and no leaf blade when beginning to flowering, blades development delayed; petiole up to 5 cm, winged, glabrescent; leaf blades lanceolate-oblong, 10–30 mm, sparely pubescent on both surfaces, abaxially furfuraceous, pinnatipartite; leaf segments 5–10 pairs, triangular-ovate to oblong-ovate, margin dentate; leafe veins sparely pubescent. Cauline leaves alternate or pseudo-opposite; petiole 0.5–5 cm, sparely pubescent; leaf blades and segments similar to basal ones. Flowers alternate and axillary, dense, flowering ± synchronous; pedicel 0.5–4 cm, sparely pubescent. Calyx tube 0.8–1 cm, 1/3–2/5 cleft anteriorly, mid-upper part inflated in flowering, sparsely long-pubescent; calyx lobes 3 or 5, rarely 2, lateral lobes leaflike, and posterior lobe ± entire or absent. Corolla purple-red, base whitish, and white spots on the base of galea and the center of lower lip; corolla tube 3–6.5 cm, slender, glabrescent, ridged; galea strongly twisted apically; beak slender, semicircular or slightly S-shaped, bent upward, to 1.2 cm; lower lip ciliate, 1.5–2.0 × 1.5–1.8 cm, lobes emarginate, middle lobe smaller and involute; filaments attached near tube throats, pubescent. Capsule obliquely oblong, apiculate, 1.4–1.7 × 0.4–0.6 cm; seed black, linear-ovate.

#### Distribution and habitat

*Pedicularis delavayi* is endemic to the Hengduan Mountains region. After re-examination of the herbarium specimens and extensive field expeditions, we confirmed that this species occurs in northwest Yunnan (Deqin, Lijiang, and Shangri-La counties), and west and north Sichuan (Daocheng, Jiulong, Kangding, Luding, Miangning, Muli, Songpan, and Xiangcheng counties). This species mainly grows in alpine meadows or at the margin of alpine shrub, at the altitude over 3600 m a.s.l.

#### Phenology

According to field collection and herbarium records, flowering individuals were collected from early June to early August. Fruiting specimens conserved at the herbaria were difficult to identify. In August 2007 and 2008, we collected fruiting individuals with mature seeds at Daxueshan Mountain of Shangri-La, northwest Yunnan.

#### Conservation status

*Pedicularis delavayi* is not common, and it is restricted to alpine meadows. Its habitats may be threatened by human activities in pasture and tourism. This species can be considered Least Concern (LC) according to IUCN Red List criteria.

#### Selected specimens examined

China. Yunnan: Deqin, *L*.*-M*. *Gao et al*., *25794* (KUN); Lijiang, Yulong Mt. *H*.*-D*. *Li & H*. *Tang LHD2014-01* (KUN), *LHD201-20* (KUN), *LHD201340* (KUN); Shangi-La, *W*.*-B*.*Yu 015* (KUN), *W*.*-B*.*Yu et al*. *HW10130* (KUN), *HW10172* (KUN), *HW10200* (KUN), *LIDZ1258* (KUN). Sichuan: Daocheng, Bowa Mt., *Sichuan Vegetation Exped*. *1923* (CDBI); Jiulong, *Qing-Quan Wang 20508* (CDBI), *W*.*-B*.*Yu et al*., *LIDZ1116* (KUN), *YWB201507224* (KUN), *YWB201507260* (KUN); Kangding, Zheduo Mt., *Ru Jiang & Cun-Li Jin 02086* (KUN, PE), W.B.Yu et al., HW10316 (KUN); Xiangcheng, Wuming Mt., *Fu-Sheng Yang Y0071* (PE).

***Pedicularis milliana*** W. B. Yu, D. Z. Li & H. Wang, sp. nov. [urn:lsid:ipni.org:names: 77185944–1] Type: China. Yunnan: Shangri-la, Xiaozhongdian, Tianbao Mountain, 27°36′22.8″N, 99°53′14.4″E, Alt. 3687m, 22 July 2010, *Wen-Bin Yu*, *Wei Jiang*, *Yang Luo & Min-Lu Liu HW10095* (holotype, KUN; isotypes: KUN). Fig 1C, S3 and S4 Figs.

Perennial herbs, low to tall, drying black or not. Roots usually cylindric. Stems solitary and ± erect, or sometimes numerous and outer stems procumbent, striate, pubescent or sparsely pubescent. Leaves basal and cauline; petiole of basal leaves 15–30 mm, petiole of cauline leaves 10–25 mm, winged, sparsely long pubescent; leaf blade lanceolate-oblong to linear-oblong, 10–60 × 7–16 mm, abaxially sparsely long pubescent along midvein, furfuraceous, adaxially glabrescent or sparsely pubescent, pinnatisect; segments 6–15 pairs, somewhat lanceolate to broadly ovate or triangular, pinnatifid, or double dentate. Flowers axillary, dense, sometimes interrupted at basal position; bracts leaflike, glabrescent or long ciliate. Calyx pubescent; tube to 1.2 cm, 1/4–1/3 cleft anteriorly; lobes 3, lateral lobes large and leaflike, posterior one smallest. Corolla rose-red; tube 40–80 mm, finely pubescent; galea strongly twisted apically, without a conspicuously auriculate protrusion; beak semicircular or slightly S-shaped, to 1.1 cm, slender; lower lip lobse 3, ciliate, 1.1–1.5 × 1.5–2.0 cm, 2 lateral lobes larger, slightly incurved at the upper margin, middle lobe slightly smaller, emarginate, 2-lobed. Anterior filament pair pubescent. Capsule ovoid-oblong, to 20 mm long; seed dark brown, linear-ovate.ca. 1.2 × 3.0 mm.

#### Distribution and habitat

*Pedicularis milliana* is endemic to northwestern Yunnan. This new species mainly occurs in humid meadows, along the grassland of mountain streams, or at the margin of low shrubs, at the altitude between 3000 m and 4000 m. Generally, this species grows close to wetland species, *P*. *longiflora*, *P*. *rhinanthoides*, and/or *P*. *cephalantha*.

#### Phenology

According to field collection and herbarium records, flowering plants were collected from early June to early August. Fruiting specimens were collected from July to September.

#### Conservation

*Pedicularis milliana* is a common species in alpine meadows in northwestern Yunnan. Its habitats are likely to be threatened by human activities. This species can be considered Least Concern (LC) according to IUCN Red List criteria.

#### Etymology

The species epithet honors Dr. Robert R. Mill, who works at the Royal Botanic Garden Edinburgh, UK. Dr. Mill is a taxonomic expert for *Pedicularis* and several other groups of seed plants, and authored dozens of papers or book chapter on the taxonomy and revision of *Pedicularis*.

#### Additional examined specimens

China. Yunnan: Deqin, *S*.*-D*. *Zhang & H*.*-J*. *He*, *08836* (KUN), *W*.*-B*. *Yu et al*. *2014123* (KUN); Lijiang, *W*.*-B*. *Yu et al*. *LIDZ1580* (KUN), *YWB-003* (KUN); Shangri-La, *W*.*-B*. *Yu et al*., *HW10095* (KUN), *HW10122* (KUN), *HW10141* (KUN), *HW10156* (KUN), *HW10163* (KUN); Weixi, *Hengduanshan Exped*. *01644*, *3104* (PE), *W*.*-B*. *Yu et al*. *2014099* (KUN).

## Supporting information

S1 Table Taxonomic overview of *P*. *delavayi*, *P*. *siphonantha* and its allies. (DOCX)

S2 Table Summary of studied species in this study, including voucher information and GenBank accessions. (XLSX)

S1 Fig Holotype of *Pedicularis delavayi* Franch. ex Maxim., *Delavay s*.*n*. (barcode LE 01010308). This photo was prepared by A. E. Grabovskaya-Borodina.
(JPG)

S2 Fig Line drawing of *Pedicularis delavayi* Franch. ex Maxim. This drawing is based on the gathering *W*.*-B*. *Yu 015* (KUN) from Daxue Mountain, Shangri-La, NW Yunnan. A, Habit. B, leave. C, calyx tube. D, calyx tube open. E, corolla lower lip. F, stamens and style. This line drawing was prepared by X-L. Wu.
(JPG)

S3 Fig Holotype of *Pedicularis milliana* W. B. Yu, D. Z. Li & H. Wang, *W*.*-B*. *Yu et al*. *HW2015095* (KUN). This gathering was collected at Tianbao Mountain, Shangri-La, NW Yunnan. This photo was taken by W.-B. Yu.
(JPG)

S4 Fig Line drawing of *Pedicularis milliana* W. B. Yu, D. Z. Li & H. Wang based on *W*.*-B*. *Yu et al*. *HW2015095* (holoype, KUN), from the left plant in S3 Fig. A. Habit; B. calyx; C. flower; D. stamens and style. This line drawing was prepared by M.-L. Liu.
(JPG)

S1 File DNA sequence matrix of the five DNA markers. The matrix is partitioned by regions.
(NEX)

## References

[1] Yu W-B, Cai J, Wang H, Chen J-Q. Advances in floral divergence and reproductive adaptation in *Pedicularis* L. (Orobanchaceae). Chinese Bulletin of Botany. 2008;25(4):392–400.

[2] Li H-L. Evolution in the flowers of *Pedicularis*. Evolution. 1951;5:158–64.

[3] Liu M-L, Yu W-B, Wang H. Rapid identification of plant species and iflora: application of DNA barcoding in a large temperate genus Pedicularis (Orobanchaceae). Plant Diversity and Resources. 2013;35(6):707–14.

[4] Kress WJ, Wurdack KJ, Zimmer EA, Weigt LA, Janzen DH. Use of DNA barcodes to identify flowering plants. P Natl Acad Sci USA. 2005;102(23):8369–74. 10.1073/pnas.0503123102 ISI:000229650500053. 15928076 PMC1142120

[5] Hebert PDN, Cywinska A, Ball SL, deWaard JR. Biological identifications through DNA barcodes. Proceedings of the Royal Society of London Series B-Biological Sciences. 2003;270(1512):313–21. 10.1098/rspb.2002.2218 ISI:000181064200013. 12614582 PMC1691236

[6] China Plant BOL Group. Comparative analysis of a large dataset indicates that internal transcribed spacer (ITS) should be incorporated into the core barcode for seed plants. P Natl Acad Sci USA. 2011;108:19641–6. 10.1073/pnas.1104551108 22100737 PMC3241788

[7] Yu W-B, Huang P-H, Ree RH, Liu M-L, Li D-Z, Wang H. DNA barcoding of *Pedicularis* L. (Orobanchaceae): evaluating four universal DNA barcoding loci in a large and hemiparasitic genus. J Sys Evol. 2011;49(5):425–37. 10.1111/j.1759-6831.2011.00154.x

[8] Yu W-B, Liu M-L, Wang H, Mill RR, Ree RH, Yang J-B, et al Towards a comprehensive phylogeny of the large temperate genus *Pedicularis* (Orobanchaceae), with an emphasis on species from the Himalaya-Hengduan Mountains. BMC Plant Biology. 2015;15(1):176 10.1186/s12870-015-0547-9 26159907 PMC4498522

[9] Ree RH. Phylogeny and the evolution of floral diversity in *Pedicularis* (Orobanchaceae). International Journal of Plant Sciences. 2005;166(4):595–613. 10.1086/430191

[10] Tkach N, Ree RH, Kuss P, Roser M, Hoffmann MH. High mountain origin, phylogenetics, evolution, and niche conservatism of arctic lineages in the hemiparasitic genus Pedicularis (Orobanchaceae). Mol Phylogenet Evol. 2014;76:75–92. 10.1016/j.ympev.2014.03.004 WOS:000336820800008. 24631857

[11] Yang F-S, Wang X-Q. Extensive length variation in the cpDNA *trnT-trnF* region of hemiparasitic *Pedicularis* and its phylogenetic implications. Plant Syst Evol. 2007;264(3–4):251–64. 10.1007/s00606-006-0510-1 ISI:000245431600008.

[12] Robart BW, Gladys C, Frank T, Kilpatrick S. Phylogeny and biogeography of North American and Asian *Pedicularis* (Orobanchaceae). Syst Bot. 2015;40(1):229–58. 10.1600/036364415X686549

[13] Maximowicz CJ. Diagnoses plantarum novarum asiaticarum. VII. Bull Acad Sci St Petersbourg. 1888;32(4):477–629.

[14] Bonati G. Contribution à l'étude du genre *Pedicularis*. Bulletin de la Société Botanique de France. 1910;57 (18):1–35.

[15] Limpricht W. Studien über die Gattung *Pedicularis*. Repert Spec Nov Regni Veg. 1924;20:161–265.

[16] Prain D. The species of *Pedicularis* of the Indian Empaire and its frontiers. Ann Roy Bot Gard Clac. 1890;3:1–196.

[17] Li H-L. A revision of the genus *Pedicularis* in China. part II. Proceedings of the Academy of Natural Sciences of Philadelphia. 1949;101:1–214.

[18] Tsoong P-C. Scrophulariaceae (Pars II) In: Chien S-S, Chun W-Y, editors. Flora Reipublicae Popularis Sinacae. 68 Beijing: Science Press; 1963 p. 1–378.

[19] Yang H-B, Holmgren NH, Mill RR. *Pedicularis* Linn In: Wu Z-Y, Raven P-H, editors. Flora of China. St. Louis, Beijing: Missouri Botanical Garden Press & Science Press; 1998 p. 97–209.

[20] Guan KY. Highland flowers of Yunnan. Kunming: Yunnan Science and Techology Press; 1998.

[21] Xu B, Li Z-M, Sun H. Seed plants of the alpine subnival belt from the Hengduan mountians, SW China. Beijing: Science Press; 2013. 413 p.

[22] Yu W-B, Zhang S-D, Wang H. New taxa of *Pedicularis* (Scrophulariaceae) from the Hengduan Mountains, Southwestern China. Novon. 2008;18(1):125–9. 10.3417/2006032

[23] Huang S-Q, Fenster CB. Absence of long-proboscid pollinators for long-corolla-tubed Himalayan *Pedicularis* species: implications for the evolution of corolla length. International Journal of Plant Sciences. 2007;168(3):325–31. ISI:000245270100006.

[24] Huang S-Q, Wang X-P, Sun S-G. Are long corolla tubes in *Pedicularis* driven by pollinator selection? Journal of Integrative Plant Biology. 2016;58(8):698–700. 10.1111/jipb.12460 26714618

[25] Yang C-F, Sun S-G, Guo Y-H. Resource limitation and pollen source (self and outcross) affecting seed production in two louseworts, *Pedicularis siphonanth*a and *P*. *longiflora* (Orobanchaceae). Bot J Linn Soc. 2005;147(1):83–9. ISI:000226700800004.

[26] Yang CF, Guo YH, Gituru RW, Sun SG. Variation in stigma morphology—How does it contribute to pollination adaptation in *Pedicularis* (Orobanchaceae)? Plant Syst Evol. 2002;236(1–2):89–98. 10.1007/s00606-002-0223-z ISI:000180234400007.

[27] Yu W-B, Cai J, Li D-Z, Mill RR, Wang H. Floral ontogeny of *Pedicularis* (Orobanchaceae), with an emphasis on the corolla upper lip. Journal of Systematics and Evolution. 2013;51(4):435–50. 10.1111/Jse.12018 WOS:000321621800007.

[28] Li R, Shi X, Yu W-B, Feng S, Sun W. Rediscovery of the supposedly extinct *Pedicularis humilis* in the eastern Himalayas. Oryx. 2016;50(02):204-. 10.1017/S0030605316000016

[29] Liu M-L, Yu W-B. *Pedicularis wanghongiae* (Orobanchaceae), a new species from Yunnan, southwestern China. Phytotaxa. 2015;217(1):53–62.

[30] Yu W-B, Huang P-H, Li D-Z, Wang H. Incongruence between nuclear and chloroplast DNA phylogenies in *Pedicularis* section *Cyathophora* (Orobanchaceae). Plos One. 2013;8(9):e74828 10.1371/journal.pone.0074828 24069353 PMC3777957

[31] Kearse M, Moir R, Wilson A, Stones-Havas S, Cheung M, Sturrock S, et al Geneious Basic: An integrated and extendable desktop software platform for the organization and analysis of sequence data. Bioinformatics. 2012;28(12):1647–9. 10.1093/bioinformatics/bts199 WOS:000305419800052. 22543367 PMC3371832

[32] Katoh K, Toh H. Parallelization of the MAFFT multiple sequence alignment program. Bioinformatics. 2010;26(15):1899–900. 10.1093/bioinformatics/btq224 20427515 PMC2905546

[33] Vaidya G, Lohman DJ, Meier R. SequenceMatrix: concatenation software for the fast assembly of multi-gene datasets with character set and codon information. Cladistics. 2011;27(2):171–80. 10.1111/j.1096-0031.2010.00329.x ISI:000288124600005. 34875773

[34] Tamura K, Stecher G, Peterson D, Filipski A, Kumar S. MEGA6: Molecular Evolutionary Genetics Analysis Version 6.0. Mol Biol Evol. 2013;30(12):2725–9. 10.1093/molbev/mst197 24132122 PMC3840312

[35] Ronquist F, Huelsenbeck JP. MrBayes 3: Bayesian phylogenetic inference under mixed models. Bioinformatics. 2003;19(12):1572–4. 10.1093/bioinformatics/btg180 ISI:000184878700016. 12912839

[36] Darriba D, Taboada GL, Doallo R, Posada D. jModelTest 2: more models, new heuristics and parallel computing. Nat Meth. 2012;9(8):772. 10.1038/nmeth.2109 PMC4594756 22847109

[37] Guindon S, Gascuel O. A simple, fast, and accurate algorithm to estimate large phylogenies by maximum likelihood. Systematic Biology. 2003;52(5):696–704. 10.1080/10635150390235520 ISI:000185732500010. 14530136

[38] Gelman A, Rubin DB. Inference from iterative simulation using multiple sequences. 1992:457–72. 10.1214/ss/1177011136

[39] Alfaro ME, Zoller S, Lutzoni F. Bayes or bootstrap? A simulation study comparing the performance of Bayesian Markov chain Monte Carlo sampling and bootstrapping in assessing phylogenetic confidence. Mol Biol Evol. 2003;20(2):255–66. 10.1093/molbev/msg028 ISI:000181036100013. 12598693

[40] Erixon P, Svennblad B, Britton T, Oxelman B. Reliability of Bayesian posterior probabilities and bootstrap frequencies in phylogenetics. Systematic Biology. 2003;52(5):665–73. . 14530133 10.1080/10635150390235485

[41] Kolaczkowski B, Thornton JW. Effects of branch length uncertainty on Bayesian posterior probabilities for phylogenetic hypotheses. Mol Biol Evol. 2007;24(9):2108–18. 10.1093/molbev/msm141 ISI:000249587200022. 17636043

[42] Stamatakis A, Hoover P, Rougemont J. A rapid bootstrap algorithm for the RAxML web servers. Systematic Biology. 2008;57(5):758–71. 10.1080/10635150802429642 ISI:000259995600008. 18853362

[43] Hillis DM, Bull JJ. An empirical test of bootstrapping as a method for assessing confidence in phylogenetic analysis. Systematic Biology. 1993;42(2):182–92. 10.1093/sysbio/42.2.182

[44] McNeil D, editor. International Code of Nomenclature for algae, fungi, and plants (Melbourne Code), adopted by the eighteenth International Botanical Congress Melbourne, Australia, July 2011 (Regnum Vegetabile, 154). Ruggell: A.R.G. Gantner Verlag; 2012.

[45] Don D, Hamilton F, Wallich N. Prodromus florae Nepalensis: sive Enumeratio vegetabilium quae in itinere per Nepaliam proprie dictam et regiones conterminas, ann. 1802–1803. Londini: J. Gale; 1825.

[46] Pennell FW. The Scrophulariaceae of eastern temperate North America. Philadelphia: The Academy of Natural Sciences of Philadelphia; 1935.

[47] Handel-Mazzetti HREv. Plantae novae sinenses. Akademie der Wissenschaften in Wien Mathematisch-Naturwissenschaftliche Klasse 1923;60:114–8.

[48] Pennell FW. The Scrophulariaceae of the Western Himalayas. The Academy of Natural Sciences of Philadelphia Monographs 1943;5:1–163.

[49] Yamazaki T. A revision of the genus Pedicularis in Nepal In: Ohba H, Malla SB, editors. The Himalayan Plants. 1 Tokyo: University Museum, University of Tokyo; 1988 p. 91–161.

[50] Husain T, Garg A, Agnihotri P. Genus Pedicularis L. (Scrophulariaceae) in India: a revisionary Study: Bishen Singh Mahendra Pal Singh; 2010.

[51] Bonati G. The alpine and subalpine vegestation of south-east Sikkim. Records of the Botanical Survey of India. 1904;4:323–431.

[52] Bonati G. New species of the genera *Phtheirospermum* and *Pedicularis*. Notes from the Royal Botanic Garden, Edinburgh. 1921;13:103–41.

[53] Mill RR. Pedicularis L. (Scrophulariaceae) In: Grierson AJC, Long DG, editors. Flora of Bhutan. 2 (3). Edinburgh: Royal Botanic Garden Edinburgh; 2001 p. 1156–234.

[54] Li H-L. A revision of the genus *Pedicularis* in China. part I. Proceedings of the Academy of Natural Sciences of Philadelphia. 1948;100:205–378.

[55] Tsoong P-C. A new system for the genus *Pedicularis*. Acta Phytotax Sin. 1955;4:71–147.

[56] Macior LW, Sood SK. Pollination ecology of *Pedicularis megalantha* D. Don (Scrophulariaceae) in the Himachal Himalaya. Plant Species Biol. 1991;6(2):75–81.

[57] Macior LW, Tang Y. A preliminary study of the pollination ecology of *Pedicularis* in the Chinese Himalaya. Plant Species Biol. 1997;12(1):1–7.

[58] Macior LW, Tang Y, Zhang J-C. Reproductive biology of *Pedicularis* (Scrophulariaceae) in the Sichuan Himalaya. Plant Species Biol. 2001;16(1):83–9.

[59] Wang H, Li D-Z. Pollination biology of four *Pedicularis* species (Scrophulariaceae) in northwestern Yunnan, China. Ann MO Bot Gard. 2005;92(1):127–38. ISI:000229456900009.

[60] Yu W-B, Li D-Z, Wang H. Highly efficient pollination by bumblebees ensures seed production in *Pedicularis lachnoglossa* (Orobanchaceae), an early-flowering Himalayan plant. J Sys Evol. 2012;50(3):218–26. 10.1111/j.1759-6831.2012.00180.x

[61] Yang CF, Wang QF. Nectarless flowers with deep corolla tubes in *Pedicularis*: does long pistil length provide an arena for male competition? Bot J Linn Soc. 2015;179(3):526–32. 10.1111/boj.12331 WOS:000364530100013.

[62] Tsoong P-C. A new system for the genus *Pedicularis* (continued II). Acta Phytotax Sin. 1956;5:205–78.

[63] Tsoong P-C. A new system for the genus *Pedicularis* (continued III). Acta Bot Sin. 1961;9(3–4):230–74.

[64] Wang H. *Pedicularis* L In: Chen S-K, Wang H, editors. Flora Yunnanica, Vol 16 Beijing: Science Press; 2006 p. 468–611.

[65] Wang W-T, Wu S-G, editors. Vascular plants of the Hengduan Mountains (Part II). Beijing: Science Press; 1994.

[66] Cai J, Wang H, Gu Z-J, Mill RR, Li D-Z. Karyotypes of thirteen species of *Pedicularis* (Orobanchaceae) from the Hengduan Mountains Region, NW Yunnan, China. Caryologia. 2004;57(4):337–47. ISI:000228079000003.

