# Supplementary material for: Phylogenetic approaches resolve taxonomical confusion in Pedicularis (Orobanchaceae): Reinstatement of Pedicularis delavayi and discovering a new species Pedicularis milliana
Source: PLoS One. 2018 Jul 25;13(7):e0200372. doi: 10.1371/journal.pone.0200372 (PMC6059426; doi:10.1371/journal.pone.0200372)
Supplement: S1 Table — (DOCX) [file pone.0200372.s001.docx]

**S1 Table.** Taxonomic overview of *P. delavayi*, *P. siphonantha* and its allies.

| **Maximowicz (1888)** | **Prain (1890)** | **Bonati (1908-1921)** | **Li (1949)** | **Tsoong (1953-1963)** | **This study** |
| --- | --- | --- | --- | --- | --- |
| ***P. siphonantha* D.Don**  ***P. delavayi* Franch. ex Maxim**  ***P. sigmoidea* Franch. ex Maxim.**  **.** | ***P. siphonantha* D.Don**  **var. *siphonantha***  **α. "vera"**  **β. "Hookeriana-himalayca"**  syn.: *P. hookeriana* Wall., *P. himalayca* Klotzsch  **var. *brevituba* Prain**  **γ. "Elephas"**  syn.: *P. elephas* Boiss.  **δ. "punctata"**  syn.: *P. punctata* Dene., *P. labellata* Boiss. (not Jacquem. ex Decne. 1844)  ***P. delavayi* Franch. ex Maxim**  ***P. sigmoidea* Franch. ex Maxim.** | ***P. siphonantha* D.Don**  **var. *siphonantha***  **var. *birmanica* Bonati**  **var. *prostrata* Bonati**  ***P. dolichantha* Bonati**  ***P. fastigiata* Franch.**  ***P. humilis* Bonati**  ***P. sigmoidea* Franch. ex Maxim.** | ***P. delavayi* Franch. ex Maxim.**  ***P. dolichantha* Bonati**  ***P. dolichosiphon* (Hand.-Mazz.) H.L.Li**  Syn.: *P. siphonantha* var. *dolichosiphon* Hand.-Mazz.  ***P. fastigiata* Franch.**  ***P. humilis* Bonati**  ***P. leptosiphon* Li**  ***P. sigmoidea* Franch. ex Maxim.**  ***P. tenuituba* Pennell & Li**  ***P. variegata* Li** | ***P. siphonantha* D.Don**  **subsp. *siphonantha***  **subsp. *prostrata* (Bonati) P.C. Tsoong**  syn: *P. siphonantha* var. *prostrata* Bonati  ***P. siphonantha* var. *delavayi* (Franch. ex Maxim.) P.C.Tsoong**  syn.: *P. delavayi* Franch. ex Maxim.  ***P. dolichantha* Bonati**  ***P. fastigiata* Franch.**  ***P. humilis* Bonati**  ***P. leptosiphon* Li**  ***P. sigmoidea* Franch. ex Maxim.**  ***P. tenuituba* Pennell & Li**  ***P. variegata* Li** | ***P. siphonantha D.Don***  **var. *siphonantha***  **var. *prostrata* Bonati**  syn: *P. siphonantha* subsp. *prostrata* (Bonati) P.C. Tsoong  ***P. delavayi* Franch. ex Maxim.**  syn.: *P. siphonantha* var. *delavayi* (Franch. ex Maxim.) P.C.Tsoong  ***P. dolichantha* Bonati**  ***P. dolichosiphon* (Hand.-Mazz.) H.L. Li**  Syn.: *P. siphonantha* var. *dolichosiphon* Hand.-Mazz.  ***P. fastigiata* Franch.**  ***P. humilis* Bonati**  syn.: *P. siphonantha* var. *birmanica* Botani (syn. nov.)  ***P. leptosiphon* Li**  ***P. sigmoidea* Franch. ex Maxim.**  ***P. tenuituba* Pennell & Li**  syn.: *P. siphonatha* var. *stictochila* W.B.Yu & H.Wang (syn. nov.)  ***P. variegata* Li** |
