# Supplementary figures and images for: Phylogenetic approaches resolve taxonomical confusion in Pedicularis (Orobanchaceae): Reinstatement of Pedicularis delavayi and discovering a new species Pedicularis milliana
Source: PLoS One. 2018 Jul 25;13(7):e0200372. doi: 10.1371/journal.pone.0200372 (PMC6059426; doi:10.1371/journal.pone.0200372)

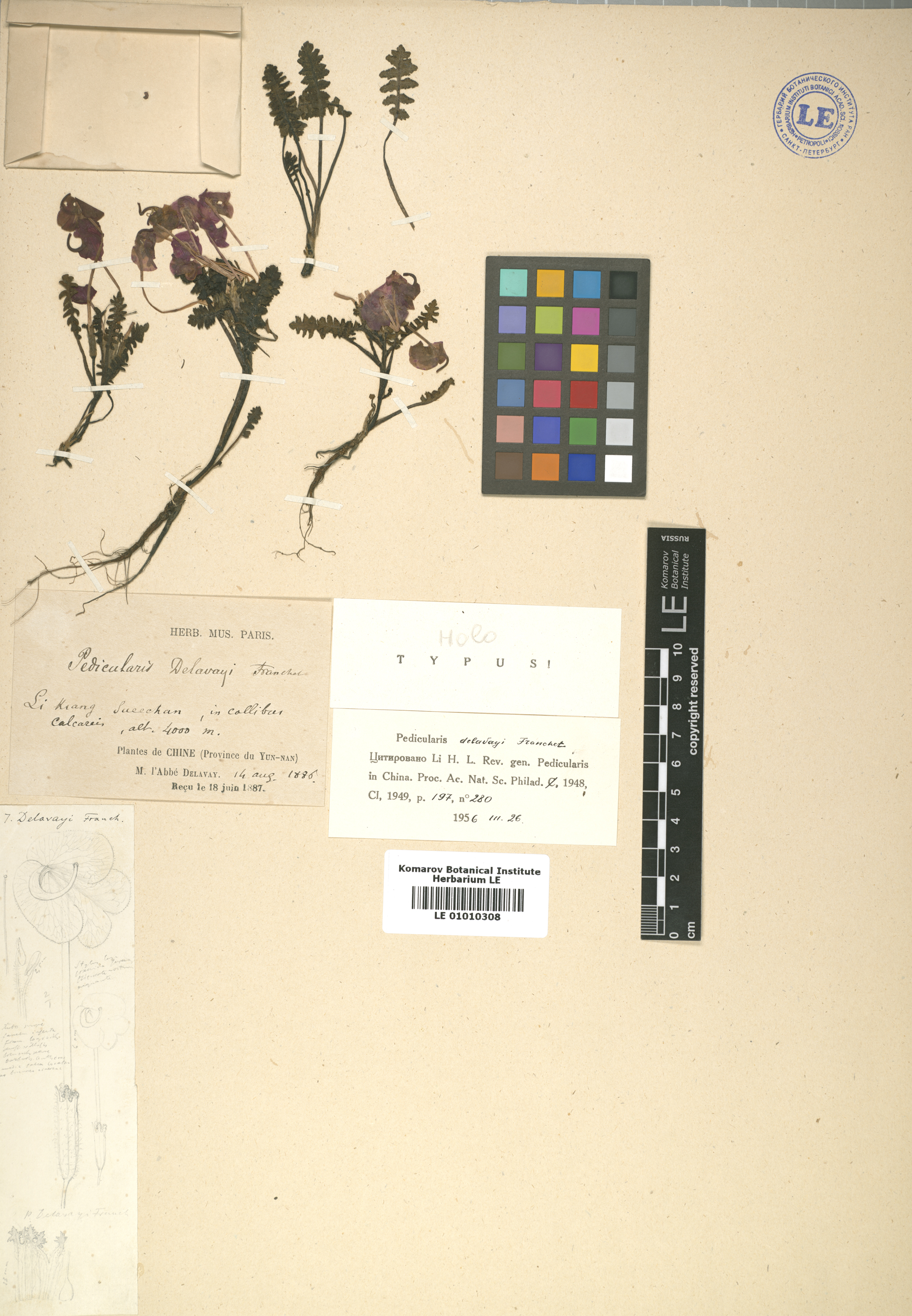

Supplement: S1 Fig — This photo was prepared by A. E. Grabovskaya-Borodina. (JPG) [file pone.0200372.s003.jpg]

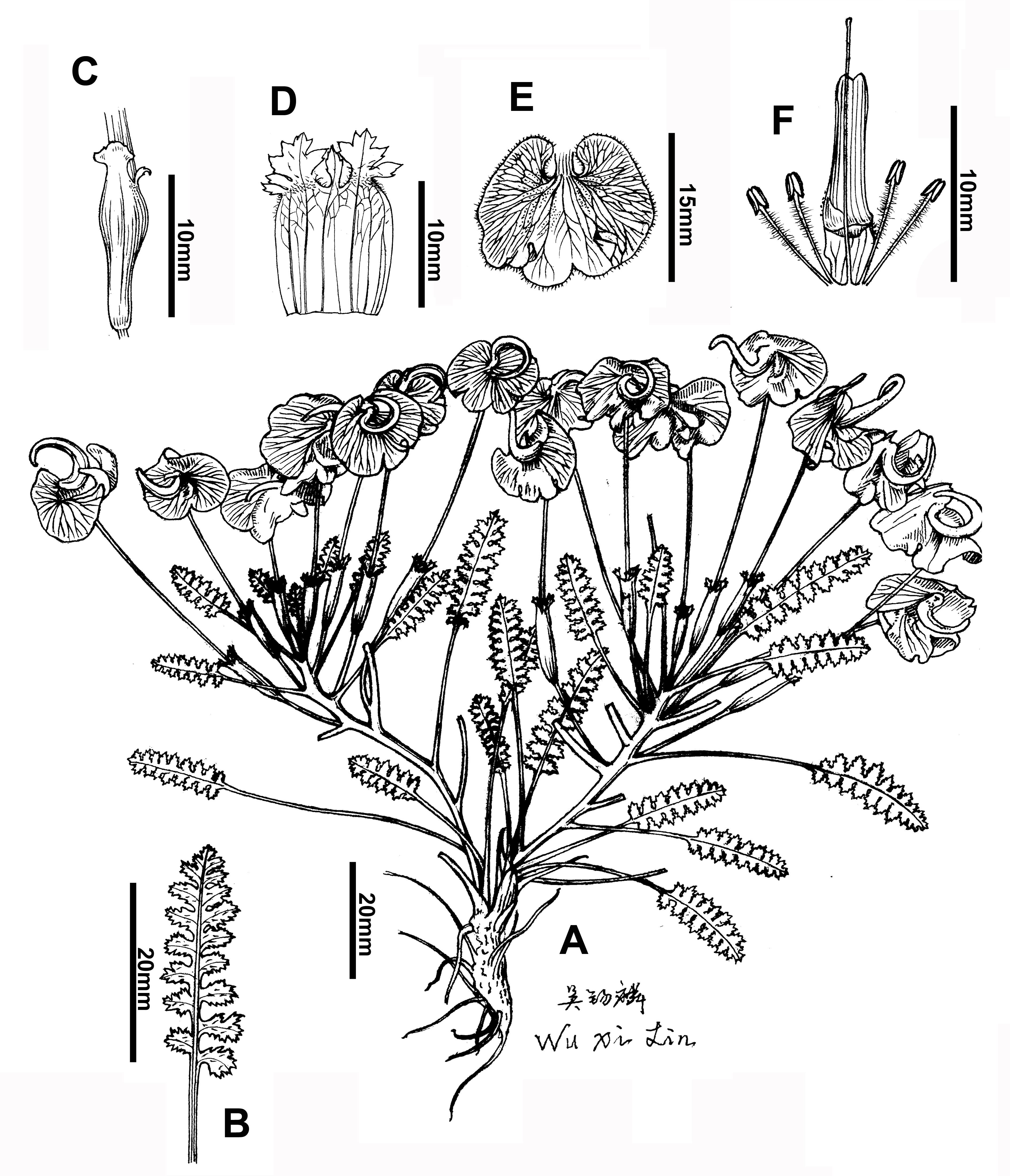

Supplement: S2 Fig — This drawing is based on the gathering W.-B. Yu 015 (KUN) from Daxue Mountain, Shangri-La, NW Yunnan. A, Habit. B, leave. C, calyx tube. D, calyx tube open. E, corolla lower lip. F, stamens and style. This line drawing was prepared by X-L. Wu. (JPG) [file pone.0200372.s004.jpg]

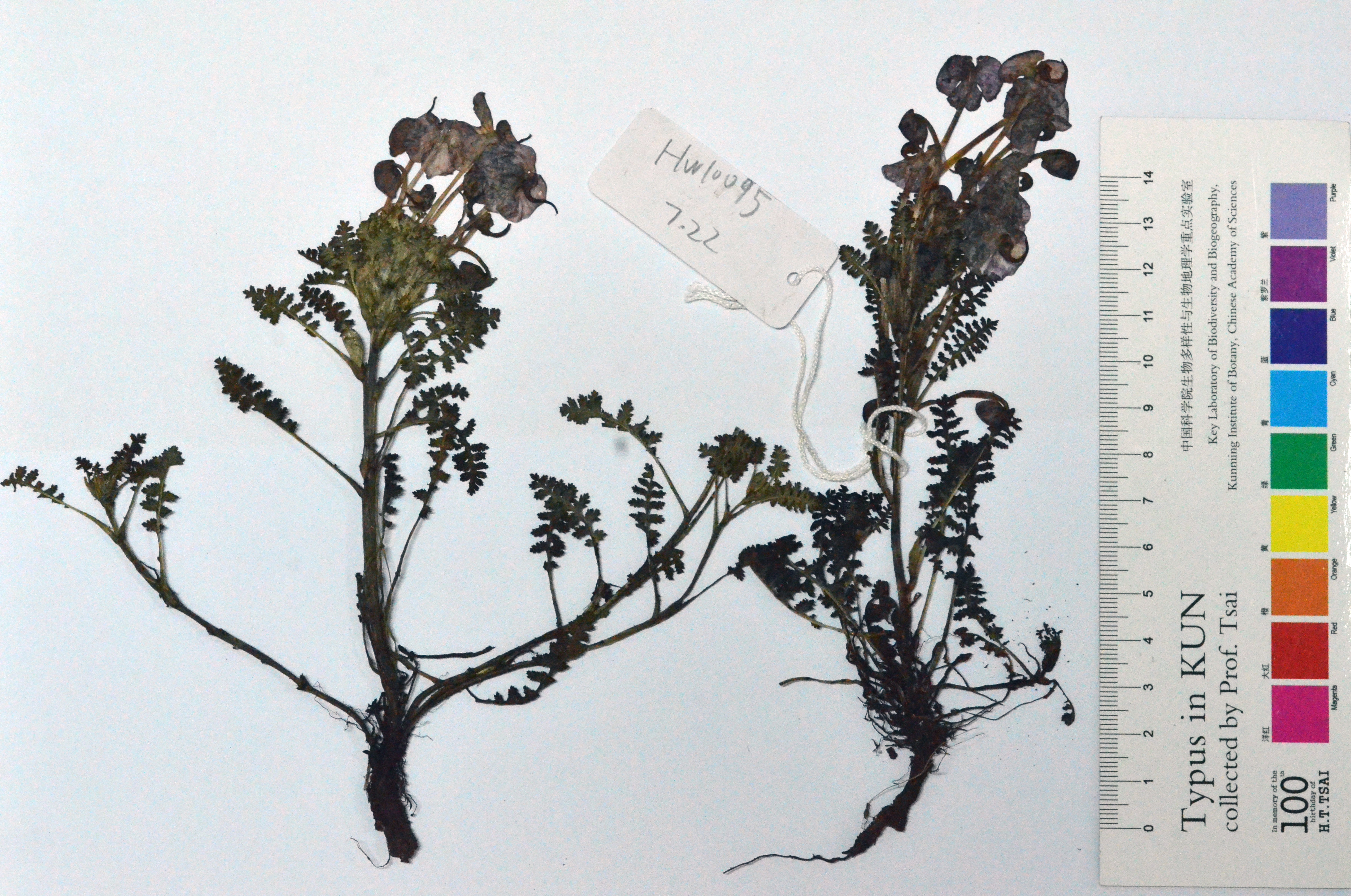

Supplement: S3 Fig — This gathering was collected at Tianbao Mountain, Shangri-La, NW Yunnan. This photo was taken by W.-B. Yu. (JPG) [file pone.0200372.s005.jpg]

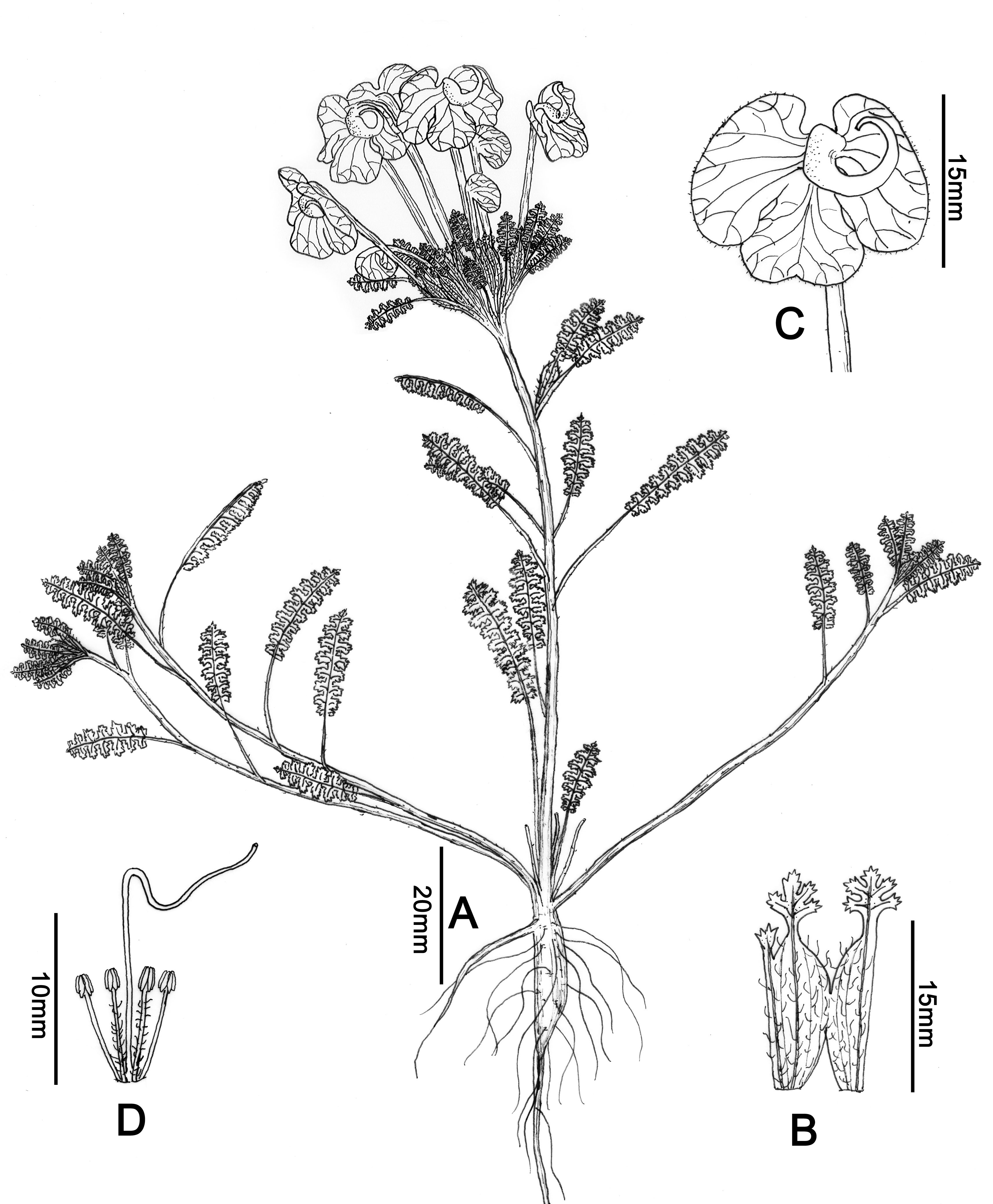

Supplement: S4 Fig — A. Habit; B. calyx; C. flower; D. stamens and style. This line drawing was prepared by M.-L. Liu. (JPG) [file pone.0200372.s006.jpg]
